# Supplementary material for: Content-rich biological network constructed by mining PubMed abstracts
Source: BMC Bioinformatics. 2004 Oct 8;5:147. doi: 10.1186/1471-2105-5-147 (PMC528731; doi:10.1186/1471-2105-5-147)
Supplement: Additional File 2 — The original results of the above study (non-essential files are deleted to keep the file size under the limit set by BMC bioinformatics). [file 1471-2105-5-147-S2.bz2 › chilibotAdditionalFile2/dip40/563ID9374547E10924/html/MST1_MST1R.html]

 


 **MST1** and **MST1R** 
  
Found 69 abstracts in PubMed, retrieved 40.  
 

 What does Google say? 
 PDF only 
| .edu only 

---

**Interactive relationship** (e.g. stimulation, inhibition, etc)

**Stimulatory relationship**- The cytokine Macrophage Stimulating 1  [ **MST1** ]   **MST1**  MSP  [ **MST1** ]  Hepatocyte Growth Factor Like  [ **MST1** ]  is a ligand of the Met related  **MST1**  Receptor  **MST1R**  RON  [ **MST1R** ] .  Ref: 12764615 Dev Genes Evol< MedlineTA>Dev Genes Evol,
- RON  [ **MST1R** ]  is a receptor tyrosine kinase activated by macrophage stimulating protein  [ **MST1** ] .  Ref: 11818458 J Leukoc Biol, 2002
- The ligand of RON  [ **MST1R** ]  was identified as macrophage stimulating protein  [ **MST1** ]  MSP  [ **MST1** ] , a member of the plasminogen related growth factor family.  Ref: 12807733 Carcinogenesis< MedlineTA>Carcinogenesis, 2002
- Macrophage stimulating protein  [ **MST1** ]  MSP  [ **MST1** ]  is a pleiotropic growth factor that signals via the Ron  [ **MST1R** ]  receptor tyrosine kinase.  Ref: 12538524 Development, 2003
- In RON  [ **MST1R** ]  expressing macrophages treated with macrophage stimulating protein  [ **MST1** ] , LPS induced prostaglandin E 2 PGE 2 production was significantly reduced.  Ref: 12177064 J Biol Chem, 2002
- Using Récepteur d Origine Nantais RON  [ **MST1R** ]  transfected cell lines, Macrophage Stimulating Protein  [ **MST1** ]  MSP  [ **MST1** ]  was identified as the ligand of RON  [ **MST1R** ] .  Ref: 9890710 Hybridoma, 1998
- Thus, RON  [ **MST1R** ]  can be activated without MSP  [ **MST1** ]  by ligand stimulation of RON  [ **MST1R** ]  associated receptors, and MSP  [ **MST1** ]  activated RON  [ **MST1R** ]  can cause ligand independent activation of RON  [ **MST1R** ]  associated receptors.  Ref: 11332718 Histol Histopathol, 2001
- The RON  [ **MST1R** ]  receptor tyrosine kinase is activated by macrophage stimulating protein  [ **MST1** ] , which regulates macrophage migration, phagocytosis, and nitric oxide production.  Ref: 12177064 J Biol Chem, 2002
- Macrophage stimulating protein  [ **MST1** ]  MSP  [ **MST1** ]  is a growth and motility factor that mediates its activity via the RON  [ **MST1R** ]  STK receptor tyrosine kinase.  Ref: 10222149 Exp Cell Res, 1999
- RON  [ **MST1R** ]  is a transmembrane receptor tyrosine kinase that mediates biological activities of Macrophage Stimulating Protein  [ **MST1** ]  MSP  [ **MST1** ] .  Ref: 11332718 Histol Histopathol, 2001
- The interaction of Ron  [ **MST1R** ]  with its ligand, known as hepatocyte growth factor like protein  [ **MST1** ]  HGFL  [ **MST1** ]  or macrophage stimulating protein  [ **MST1** ]  MSP  [ **MST1** ] , induces crucial cellular responses including invasive growth, proliferation, cell scattering, and branching morphogenesis.  Ref: 11593422 Oncogene, 2001
- RON  [ **MST1R** ]  recepteur d origine nantais is a receptor tyrosine kinase expressed in murine peritoneal resident macrophages and activated by macrophage stimulating protein  [ **MST1** ]  MSP  [ **MST1** ] .  Ref: 9794431 J Immunol, 1998
- We recently showed that macrophage stimulating protein  [ **MST1** ]  MSP  [ **MST1** ] , a serum protein homologous to hepatocyte growth factor, promotes ciliary motility by activating its receptor, RON  [ **MST1R** ] , on the airway ciliated epithelium.  Ref: 10955755 Respir Med, 2000
- The RON  [ **MST1R** ]  receptor type tyrosine kinase, a member of the hepatocyte growth factor receptor family, is a receptor for macrophage stimulating protein  [ **MST1** ]  MSP  [ **MST1** ] .  Ref: 10336478 J Biol Chem, 1999
- Recently, we established IL 3 dependent Ba F3 pro B cells ectopically expressing RON  [ **MST1R** ]  tyrosine kinase, a receptor for macrophage stimulating protein  [ **MST1** ]  MSP  [ **MST1** ] , and showed that MSP  [ **MST1** ]  stimulation specifically promoted cell morphological changes through tyrosine phosphorylation of the IL 3 common beta chain receptor subunit betac by activated RON  [ **MST1R** ]  kinase without activation of JAK2 tyrosine kinase.  Ref: 12270548 Immunol Lett, 2002
**Neutral relationship**- MSP  [ **MST1** ]  induces biosynthesis of Ron  [ **MST1R** ]  receptor in the motoneuron somata.  Ref: 11359926 Mol Biol Cell, 2001
- The specific receptor for MSP  [ **MST1** ]  is the RON  [ **MST1R** ]  recepteur d origine nantais receptor tyrosine kinase a member of the MET proto oncogene family.  Ref: 12472665 Scand J Immunol, 2002
- Moreover, GSNO or SNAP inhibited the macrophage stimulating protein  [ **MST1** ]  induced RON  [ **MST1R** ]  phosphorylation and macrophage migration.  Ref: 10725742 J Immunol, 2000
- Activation of CR3 mediated phagocytosis by MSP  [ **MST1** ]  requires the RON  [ **MST1R** ]  receptor, tyrosine kinase activity, phosphatidylinositol 3 kinase, and protein kinase C zeta.  Ref: 12773513 J Leukoc Biol, 2003
- AKT kinase is a component of a separate branch of the RON  [ **MST1R** ]  PI3 K pathway that mediates the MSP  [ **MST1** ]  anti apoptotic effect on epithelial cells.  Ref: 10080538 J Leukoc Biol, 1999
- The MSP  [ **MST1** ]  receptor is a transmembrane tyrosine kinase called RON  [ **MST1R** ]  in humans and STK in mice.  Ref: 10080538 J Leukoc Biol, 1999
- MSP  [ **MST1** ]  is the ligand for Ron  [ **MST1R** ] , a member of the Met subfamily of tyrosine kinase receptors.  Ref: 11704649 Br J Pharmacol, 2001
- These results indicate a novel field of operation for MSP  [ **MST1** ]  and suggest a pathogenic role of the MSP  [ **MST1** ]  RON  [ **MST1R** ]  system in renal disease.  Ref: 11856768 J Am Soc Nephrol, 2002
- Consistently, the mature Ron  [ **MST1R** ]  protein is expressed by the MSP  [ **MST1** ]  responsive cells but not by the unresponsive monocytes.  Ref: 11704649 Br J Pharmacol, 2001
- MSP  [ **MST1** ]  binding to RON  [ **MST1R** ]  causes receptor tyrosine phosphorylation leading to up regulation of RON  [ **MST1R** ]  catalytic activity and subsequent activation of downstream signaling molecules.  Ref: 11332718 Histol Histopathol, 2001
- MSP  [ **MST1** ]  mediates its biological activities by activating a transmembrane receptor tyrosine kinase called RON  [ **MST1R** ]  in humans or SKT in mice.  Ref: 11388667 Apoptosis, 2001
- MSP  [ **MST1** ]  in HK2 supernatant and rMSP induced in HMC phosphorylation of RON  [ **MST1R** ] , growth, migration, invasion, and IL 6 synthesis.  Ref: 11856768 J Am Soc Nephrol, 2002
- HGF Scatter factor 1 and MSP  [ **MST1** ]  Scatter Factor 2 bind to tyrosine kinase receptors encoded by the proto oncogenes MET and RON  [ **MST1R** ] .  Ref: 10871856 Oncogene, 2000
- Ron  [ **MST1R** ]  is a receptor tyrosine kinase that is activated by the binding of hepatocyte growth factor like  [ **MST1** ]  HGFL  [ **MST1** ]  protein.  Ref: 12606483 Biol Reprod, 2003
- Recently, we observed that MSP  [ **MST1** ]  induces morphological changes in interleukin IL 3 dependent Ba F3 cells ectopically expressing RON  [ **MST1R** ] .  Ref: 10336478 J Biol Chem, 1999
- We show that MSP  [ **MST1** ]  induced complement mediated phagocytosis requires the RON  [ **MST1R** ]  receptor tyrosine kinase and the alphaMbeta2 integrin, as evidenced by the inability of RON  [ **MST1R** ]  and alphaM peritoneal macrophages to augment phagocytosis of complement coated sheep erythrocytes in response to MSP  [ **MST1** ] .  Ref: 12773513 J Leukoc Biol, 2003
- The Ron  [ **MST1R** ]  STK receptor tyrosine kinase is a member of the c Met family of receptors and is activated by hepatocyte growth factor like protein  [ **MST1** ]  HGFL  [ **MST1** ] .  Ref: 10225971 J Clin Invest, 1999
- To begin to examine the involvement of HGF and MSP  [ **MST1** ]  in healing of cutaneous wounds we have mapped the temporal expression of these two molecules and their receptors, MET and RON  [ **MST1R** ]  respectively, in adult rat excisional wounds.  Ref: 11702235 Cell Tissue Res, 2001
- In these cells, MSP  [ **MST1** ]  induced RON  [ **MST1R** ]  phosphorylation concomitant with reduced iNOS mRNA expression and protein synthesis.  Ref: 9794431 J Immunol, 1998
- The blocking of MSP  [ **MST1** ]  binding to RON  [ **MST1R** ]  by the anti RON  [ **MST1R** ]  antibodies was confirmed by inhibition of cell migration induced by MSP  [ **MST1** ]  in HT 29 D4 cells.  Ref: 9890710 Hybridoma, 1998

**Non-interactive relationship** (e.g. studied together, co-existance, homology, etc.)

- Ron  [ **MST1R** ]  the receptor for Macrophage Stimulating Protein  [ **MST1** ]  has never been implicated before in human malignancies or in cell transformation.  Ref: 9715276 Oncogene, 1998
- To investigate whether MSP  [ **MST1** ]  is also capable of acting as a neurotrophic factor, we purified hypoglossal motoneurons from the embryonic chicken hindbrain because these neurons are known to express the MSP  [ **MST1** ]  receptor tyrosine kinase RON  [ **MST1R** ] .  Ref: 11860510 Eur J Neurosci, 2002
- Thus, MSP  [ **MST1** ]  and RON  [ **MST1R** ]  are potential regulators that control macrophage activities during bacterial infection in vivo.  Ref: 12472665 Scand J Immunol, 2002
- In normal human kidney, tubules expressed MSP  [ **MST1** ]  and RON  [ **MST1R** ] .  Ref: 11856768 J Am Soc Nephrol, 2002
- In this study, we demonstrate that stimulation of murine peritoneal macrophages with MSP  [ **MST1** ]  results in the RON  [ **MST1R** ]  dependent up regulation of arginase, an enzyme associated with alternative activation that competes with iNOS for the substrate L arginine, the products of which are involved in cell proliferation and matrix synthesis.  Ref: 11777982 J Immunol, 2002
- For example, MSP  [ **MST1** ]  induced cell shape change is mediated via RON  [ **MST1R** ]  activated IL 3 betac receptor.  Ref: 11332718 Histol Histopathol, 2001
- Our lab and others have previously shown that macrophage stimulating protein  [ **MST1** ]  MSP  [ **MST1** ] , through activation of its receptor RON  [ **MST1R** ] , negatively regulates NO production in response to IFN gamma and LPS by inhibiting the expression of inducible NO synthase iNOS.  Ref: 11777982 J Immunol, 2002
- The specific cellular localization and mechanisms of action of Ron  [ **MST1R** ]  and HGFL  [ **MST1** ]  during embryo implantation are not known.  Ref: 12606483 Biol Reprod, 2003
- Ron  [ **MST1R** ] , the receptor tyrosine kinase RTK for the macrophage stimulating protein  [ **MST1** ]  MSP  [ **MST1** ] , activates multiple signaling pathways by recruiting several positive regulators to a multifunctional docking site.  Ref: 12802274 Oncogene, 2003
- Kinases involved in MSP  [ **MST1** ]  RON  [ **MST1R** ]  signaling.  Ref: 10080538 J Leukoc Biol, 1999
- HMC expressed RON  [ **MST1R** ]  but did not produce MSP  [ **MST1** ] .  Ref: 11856768 J Am Soc Nephrol, 2002
- Because both cells have roles in tissue injury, we looked for active MSP  [ **MST1** ]  and expressed RON  [ **MST1R** ]  in wounds.  Ref: 9764835 J Invest Dermatol, 1998
- The overexpression of bcl 2, but not p53 in oncocytoma, suggests that the MSP  [ **MST1** ]  Ron  [ **MST1R** ]  system sustains the growth of oncocytoma by opposing apoptosis.  Ref: 12766581 Am J Surg Pathol, 2003
- Activation of the RON  [ **MST1R** ]  receptor tyrosine kinase by macrophage stimulating protein  [ **MST1** ]  inhibits inducible cyclooxygenase 2 expression in murine macrophages.  Ref: 12177064 J Biol Chem, 2002
- The expression of the MSP  [ **MST1** ]  receptor, RON  [ **MST1R** ] , was evaluated in HMC and HK2 by Western blot.  Ref: 11856768 J Am Soc Nephrol, 2002
- Activation of the stem cell derived tyrosine kinase  [ **MST1R** ]  RON  [ **MST1R** ]  receptor tyrosine kinase by macrophage stimulating protein  [ **MST1** ]  results in the induction of arginase activity in murine peritoneal macrophages.  Ref: 11777982 J Immunol, 2002
- Here we present a chimeric cytokine derived from the alpha chains of HGF and MSP  [ **MST1** ] , named Metron factor 1 for its ability to concomitantly activate the HGF receptor Met and the MSP  [ **MST1** ]  receptor Ron  [ **MST1R** ] .  Ref: 11981563 Nat Biotechnol, 2002
- Cleavage yields active MSP  [ **MST1** ] , the ligand for a membrane receptor RON  [ **MST1R** ]  that is expressed on keratinocytes as well as macrophages.  Ref: 9764835 J Invest Dermatol, 1998
- These observations suggest that activation of the Ron  [ **MST1R** ]  receptor by HGFL  [ **MST1** ]  binding may aid in implantation by way of trophoblast function and viability.  Ref: 12606483 Biol Reprod, 2003
- Activation of RON  [ **MST1R** ]  by MSP  [ **MST1** ]  results in cell migration, shape change, and proliferation.  Ref: 9890710 Hybridoma, 1998
- The proto oncogene product Ron  [ **MST1R** ]  is the receptor for macrophage stimulating protein  [ **MST1** ] , a scatter factor that stimulates cell proliferation, prevents apoptosis, and induces an invasive cell phenotype.  Ref: 12766581 Am J Surg Pathol, 2003
- The expression of MSP  [ **MST1** ]  and RON  [ **MST1R** ]  in normal human renal tissue was studied by immunohistochemistry.  Ref: 11856768 J Am Soc Nephrol, 2002
- Thus, occupancy of the RON  [ **MST1R** ]  receptor by MSP  [ **MST1** ]  activates distinct signal transduction pathways that mediate several cellular responses.  Ref: 10080538 J Leukoc Biol, 1999
- Thus, increased expression of HGF, MSP  [ **MST1** ]  and their receptors MET and RON  [ **MST1R** ]  respectively was observed in response to wounding.  Ref: 11702235 Cell Tissue Res, 2001
- This receptor collaboration extends the spectrum of cellular responses generated by MSP  [ **MST1** ]  and by putative ligands of RON  [ **MST1R** ]  associated receptors.  Ref: 11332718 Histol Histopathol, 2001
- The immunoreactivity for RON  [ **MST1R** ]  was observed in the epithelium of the esophagus, small intestine, colon, hepatocytes, Kupffer cells, and splenic macrophages both in the adult and the fetus, suggesting that the MSP  [ **MST1** ]  RON  [ **MST1R** ]  signaling pathway possesses the proper biological properties to possibly be involved in morphogenesis or differentiation of cells in these organs and cell types.  Ref: 11281194 Dig Dis Sci, 2001
- Furthermore, targeted deletion of the receptor for MSP  [ **MST1** ] , stem cell derived tyrosine kinase  [ **MST1R** ]  receptor STK RON  [ **MST1R** ] , resulted in increased production of NO by activated macrophages both in vitro and in vivo.  Ref: 10586055 J Immunol, 1999
- Although  **MST1**  deficient mice are viable,  **MST1R**  is essential in mice before gastrulation for implantation, and is a known oncogene in man.  Ref: 12764615 Dev Genes Evol< MedlineTA>Dev Genes Evol, 1999
- To investigate the functional involvement of MSP  [ **MST1** ]  and RON  [ **MST1R** ]  in bronchiectasis, in which mucociliary clearance MCC is impaired, first we examined RON  [ **MST1R** ]  expression on the bronchial ciliated epithelium of patients with bronchiectasis.  Ref: 10955755 Respir Med, 2000
- Macrophage stimulating protein  [ **MST1** ]  and RON  [ **MST1R** ]  receptor tyrosine kinase potential regulators of macrophage inflammatory activities.  Ref: 12472665 Scand J Immunol, 2002
- Oncogenic and invasive potentials of human macrophage stimulating protein  [ **MST1** ]  receptor, the RON  [ **MST1R** ]  receptor tyrosine kinase.  Ref: 12807733 Carcinogenesis< MedlineTA>Carcinogenesis, 2002
- Macrophage stimulating protein  [ **MST1** ]  MSP  [ **MST1** ]  and its receptor, RON  [ **MST1R** ] , stimulate human osteoclast activity but not proliferation effect of MSP  [ **MST1** ]  distinct from that of hepatocyte growth factor.  Ref: 9766449 Exp Hematol, 1998
- Treatment of RON  [ **MST1R** ]  expressing cells with PD98059 or U0126, inhibitors of mitogen activated protein kinase kinase activity, blocked both IL 3 and MSP  [ **MST1** ]  induced morphological changes.  Ref: 12270548 Immunol Lett, 2002
- These results clearly indicate that hrMSP induces the respiratory burst in human macrophages but not in monocytes, suggesting for the MSP  [ **MST1** ]  Ron  [ **MST1R** ]  complex a role of activator as well as of possible marker for human mature macrophages.  Ref: 11704649 Br J Pharmacol, 2001
- Activation of integrins causes MSP  [ **MST1** ]  independent RON  [ **MST1R** ]  phosphorylation, and the integrin RON  [ **MST1R** ]  collaboration regulates cell survival.  Ref: 11332718 Histol Histopathol, 2001
- Oncogenic activation of the Ron  [ **MST1R** ]  tyrosine kinase Macrophage Stimulating Protein  [ **MST1** ]  receptor relies on substitutions of two highly conserved residues in the catalytic domain D1232V and M1254T, which result in ligand independent activation of the receptor, in vivo tumorigenesis and metastasis.  Ref: 11064459 Oncogene, 2000
- Activation of RON  [ **MST1R** ]  by MSP  [ **MST1** ]  in mature exudate macrophages inhibits LPS and IFN gamma induced iNOS synthesis.  Ref: 9794431 J Immunol, 1998
- Activation of RON  [ **MST1R** ]  by MSP  [ **MST1** ]  exerts dual functions on macrophages.  Ref: 12472665 Scand J Immunol, 2002
- Immunofluorescent staining revealed both MSP  [ **MST1** ]  and RON  [ **MST1R** ]  within the wound, with maximum staining occurring between 7 and 21 days for both the ligand and receptor.  Ref: 11702235 Cell Tissue Res, 2001
- Ligand binding experiments showed that both antibodies are able to block the binding of radiolabeled MSP  [ **MST1** ]  to RON  [ **MST1R** ]  and showed also that the antibodies recognize two different epitopes in the molecule.  Ref: 9890710 Hybridoma, 1998
- HK2 produced MSP  [ **MST1** ]  and expressed RON  [ **MST1R** ]  in a form that was phosphorylated by rMSP.  Ref: 11856768 J Am Soc Nephrol, 2002
- We investigated the pathway involved in integrin mediated RTK activation, using RON  [ **MST1R** ] , the receptor for macrophage stimulating protein  [ **MST1** ] .  Ref: 10747844 J Biol Chem, 2000
- In this study we examined the role of MSP  [ **MST1** ]  RON  [ **MST1R** ]  in the signal pathway of human osteoclasts.  Ref: 9766449 Exp Hematol, 1998
